# Rectal Prolapse Associated With Chronic Ketamine Misuse: A Report of Two Cases

**DOI:** 10.7759/cureus.114526

**Published:** 2026-08-14

**Authors:** Mariam A Omar, Muhammad Ishfaq, Rajasundram Rajaganeshan, Michael S Floyd

**Affiliations:** 1 General Surgery, Whiston Hospital, Mersey and West Lancashire Teaching Hospitals NHS Trust, Prescot, GBR; 2 Urology, Whiston Hospital, Mersey and West Lancashire Teaching Hospitals NHS Trust, Prescot, GBR

**Keywords:** ketamine-induced cystitis, ketamine-induced uropathy, pelvic floor dysfunction, pelvic floor dyssynergia, rectal prolapse

## Abstract

Chronic ketamine misuse is a well-recognized cause of ketamine-induced uropathy (KIU), characterized by severe lower urinary tract symptoms and markedly reduced bladder capacity. Its potential association with anorectal dysfunction remains poorly described. We report two young adults with KIU who developed progressive pelvic floor dysfunction and anorectal pathology ranging from pelvic floor dyssynergia and internal rectal intussusception to full-thickness rectal prolapse (RP). Both patients experienced severe urinary frequency, suprapubic pain, and chronic straining to void. One patient developed solitary rectal ulcer syndrome with Grade III internal rectal intussusception, while the other developed full-thickness RP requiring colorectal assessment. These two case reports suggest a possible association between chronic ketamine misuse, severe urinary tract dysfunction, and secondary pelvic floor strain with subsequent anorectal pathology. Clinicians managing KIU should be alerted to evolving anorectal symptoms, and recreational ketamine use should be considered in young adults presenting with RP.

## Introduction

Ketamine is a dissociative anesthetic used therapeutically for procedural sedation, analgesia for burn victims, and treatment of refractory depression [1]. However, its psychotropic effects and relative affordability have led to widespread recreational misuse, particularly among adolescents and young adults. In England and Wales, recent data suggest that ketamine use has reached its highest recorded levels in recent years, with the greatest prevalence observed in individuals aged 16-24 [2]. Increasing rates of illegal ketamine consumption have been paralleled by the recognition of a spectrum of ketamine-associated systemic complications [3,4].

Ketamine-associated uropathy is among the most well-described sequelae and is characterized by severe storage lower urinary tract symptoms, including frequency, urgency, dysuria, hematuria, and suprapubic pain. Progressive inflammation and bladder wall fibrosis result in markedly reduced bladder capacity, poor compliance, vesicoureteral reflux, hydroureteronephrosis, and eventually renal impairment. Patients frequently report persistent straining to void in response to painful storage symptoms and incomplete bladder emptying, thereby generating repeated increases in intra-abdominal pressure [3,5].

Beyond the urinary tract, ketamine misuse has been associated with hepatobiliary dysfunction, such as deranged liver enzymes and biliary duct dilatation, similar to primary biliary cirrhosis. Upper GI pathology, such as gastritis and peptic ulceration, and reports of lower GI inflammation and mucosal ulceration are now emerging [6-8]. Neuropsychiatric sequelae, including cognitive impairment and memory deficits, are also well documented [9].

Several pathophysiological mechanisms have been proposed to explain these complications. It has been suggested that ketamine, as an N-methyl-D-aspartate receptor antagonist, might act on local smooth muscle or the central nervous system to affect motility and cause cramping pain. Microvascular damage caused by ketamine metabolites has been implicated in ketamine uropathy. Similar mechanisms may also underlie the GI irritation and ulceration seen in ketamine abusers. Furthermore, circulating ketamine may also precipitate unknown autoimmune responses that produce the inflammation seen in the urinary and GI tracts [7,10].

Rectal prolapse (RP) is defined as the full-thickness protrusion of the rectum through the anal canal. It may occur independently or alongside other pelvic prolapses. RP is generally understood to evolve along a continuum, from internal rectal intussusception, often detectable only on defecating proctography, to external prolapse, a progression formalized by the Oxford classification [11]. In adults, RP is thought to result from progressive failure of pelvic support structures combined with structural and functional changes in the distal colon and pelvic floor. These abnormalities, particularly when compounded by repetitive mechanical insults such as chronic straining, can facilitate intussusception and eventual full-thickness prolapse [11-13]. Clinically, RP may present with fecal incontinence, obstructed defecation, rectal bleeding, mucoid discharge, and significant psychosocial morbidity [11].

Although widely recognized in the elderly and pediatric populations, RP is rare in young adults; as such, the literature base in this group is limited. From the available studies, several risk factors have been identified. These include repetitive straining behaviors, usually associated with constipation and functional defecation disorders; chronic psychiatric illness with long-term medication use; and certain inflammatory bowel pathologies [13].

Given the central role of chronic straining in the pathogenesis of RP, it is biologically plausible that persistent urinary straining secondary to ketamine-induced bladder dysfunction may predispose susceptible individuals to progressive pelvic floor failure. The sustained Valsalva-type effort required to void in the context of a contracted, painful bladder can replicate the pathophysiological forces observed in chronic constipation, thereby facilitating the development of RP.

To our knowledge, RP occurring in the setting of chronic ketamine misuse has not previously been formally reported. We present two cases of young adults with ketamine-associated uropathy who developed a spectrum of anorectal pathology ranging from pelvic floor dyssynergia and rectoanal intussusception to full-thickness RP. Together, these cases suggest a potential mechanistic link between chronic ketamine-induced urinary dysfunction, sustained straining behavior, and progressive pelvic floor collapse.

## Case presentation

Case 1

A 20-year-old woman with a history of anxiety and depression presented to the ED with a six-week history of dysuria and suprapubic pain in December 2021. Blood tests and urine culture were unremarkable. She reported a two-year history of regular ketamine abuse, which began during the COVID-19 lockdown period and had escalated to daily use in the preceding six to seven weeks. She was referred to the alcohol and drug liaison service and to urology with suspected ketamine cystitis.

In January 2022, she was reviewed in a specialist ketamine uropathy clinic, reporting urinary frequency, dribbling, and persistent suprapubic cramping pain. These symptoms led to repeated straining to void. Cystoscopy under sedation was arranged but not tolerated, and she subsequently disengaged from follow-up. She re-presented to urology in June 2023. Renal tract ultrasound was normal. Repeat cystoscopy under general anesthesia demonstrated a bladder capacity of 300 mL. She was counseled regarding the harms of continued ketamine use and commenced on mirabegron and solifenacin for her symptoms while awaiting rehabilitation.

There was no documented history of chronic constipation, obstructed defecation, or other bowel dysfunction prior to the onset of her ketamine-associated urinary symptoms. Against a background of chronic straining to void, she developed new lower GI symptoms. In September 2023, she presented to her general practitioner with painful defecation and bloody mucoid discharge per rectum. Fecal calprotectin and celiac screening were unremarkable. In January 2024, while undergoing rehabilitation, she underwent upper and lower GI endoscopy for newly identified iron deficiency anemia. Colonoscopy revealed a solitary rectal ulcer. Histology demonstrated mild active chronic inflammation without dysplasia or malignancy. This ulcer was attributed to chronic straining behavior secondary to ketamine-related urinary dysfunction.

Her symptoms progressed to obstructed defecation requiring manual evacuation. She was referred to the pelvic floor service in September 2024 and commenced on regular laxatives and pelvic floor physiotherapy and was advised on behavioral techniques, including diaphragmatic breathing.

**Figure 1:**
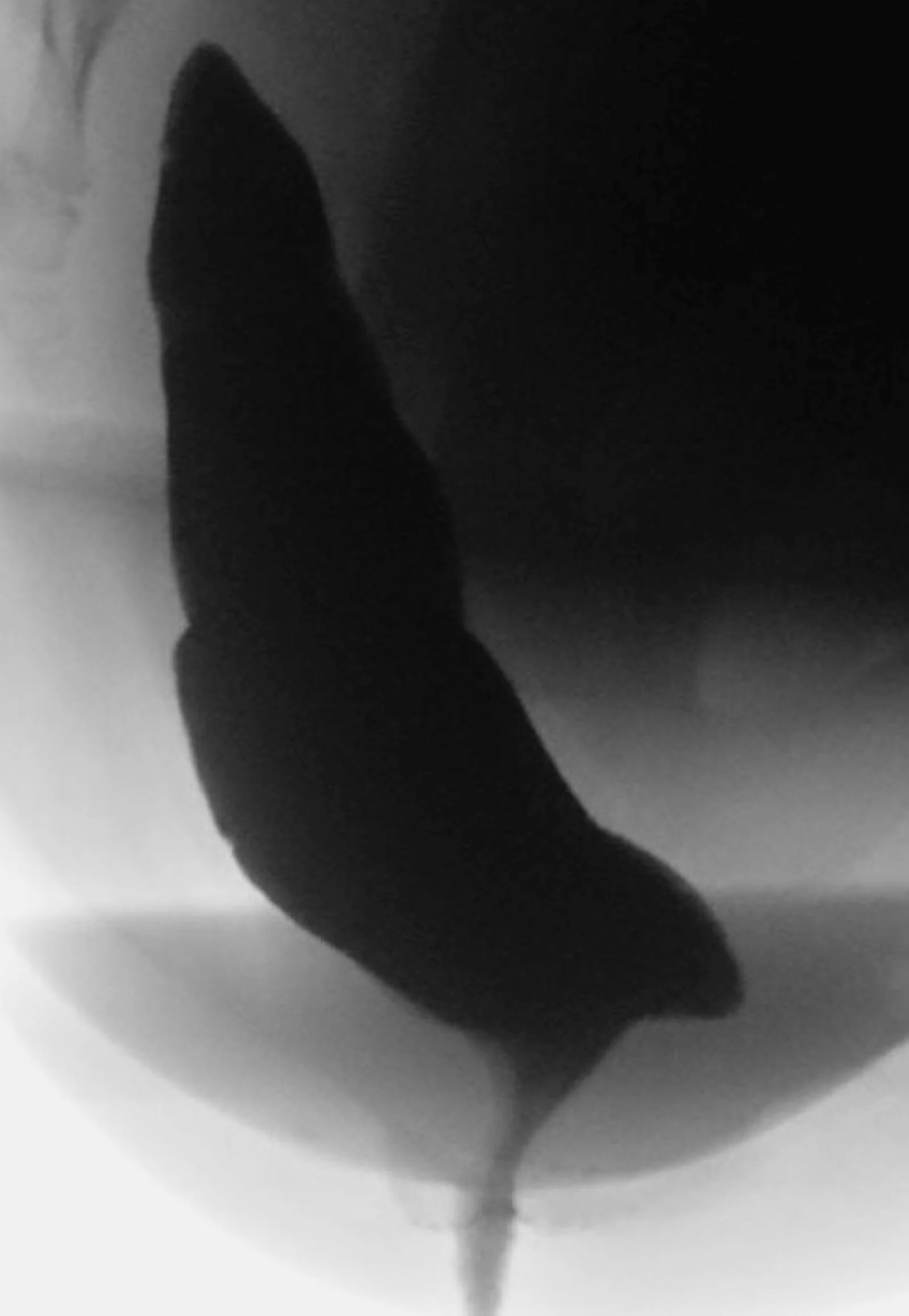
Defecating proctography demonstrating Grade III rectoanal intussusception.

In January 2025, anorectal manometry demonstrated impaired rectal propulsion with a paradoxical increase in distal anal canal pressure during attempted defecation, consistent with anismus. Defecating proctography showed Grade III rectoanal intussusception extending to the upper anal canal and a small-to-moderate anterior rectocele (Figure 1). These findings were consistent with pelvic floor dysfunction secondary to repeated straining behaviors. Ongoing physiotherapy and consideration of biofeedback therapy were recommended; however, compliance was poor, and the patient was subsequently discharged from the pelvic floor service due to repeated nonattendance.

Case 2

A 27-year-old previously fit and well man presented to the ED in June 2024 with suprapubic pain and hematuria. Initial investigations demonstrated normal renal function, inflammatory markers, and urine culture. Mildly elevated liver transaminases were noted. He revealed a four-year history of ketamine abuse, with use exceeding four times per week and averaging around 1-2 g daily.

He was referred to urology in August 2024, and CT demonstrated bilateral hydroureter with mild bilateral hydronephrosis. At his initial urology review, he reported persistent urinary frequency, urgency, and chronic suprapubic pain, exacerbated by repeated straining to void. He was counseled regarding ongoing ketamine misuse and commenced on solifenacin. Further imaging with contrast-enhanced CT and rigid cystoscopy with bladder biopsy were arranged while awaiting rehabilitation. Cystoscopy demonstrated a markedly reduced bladder capacity of 100 mL.

At follow-up in May 2025, he had abstained from ketamine for four months but continued to experience significant suprapubic pain and storage lower urinary tract symptoms. Analgesic strategies were escalated, and he was referred to the pain team. He also reported ongoing follow-up under colorectal surgery for RP.

**Figure 2:**
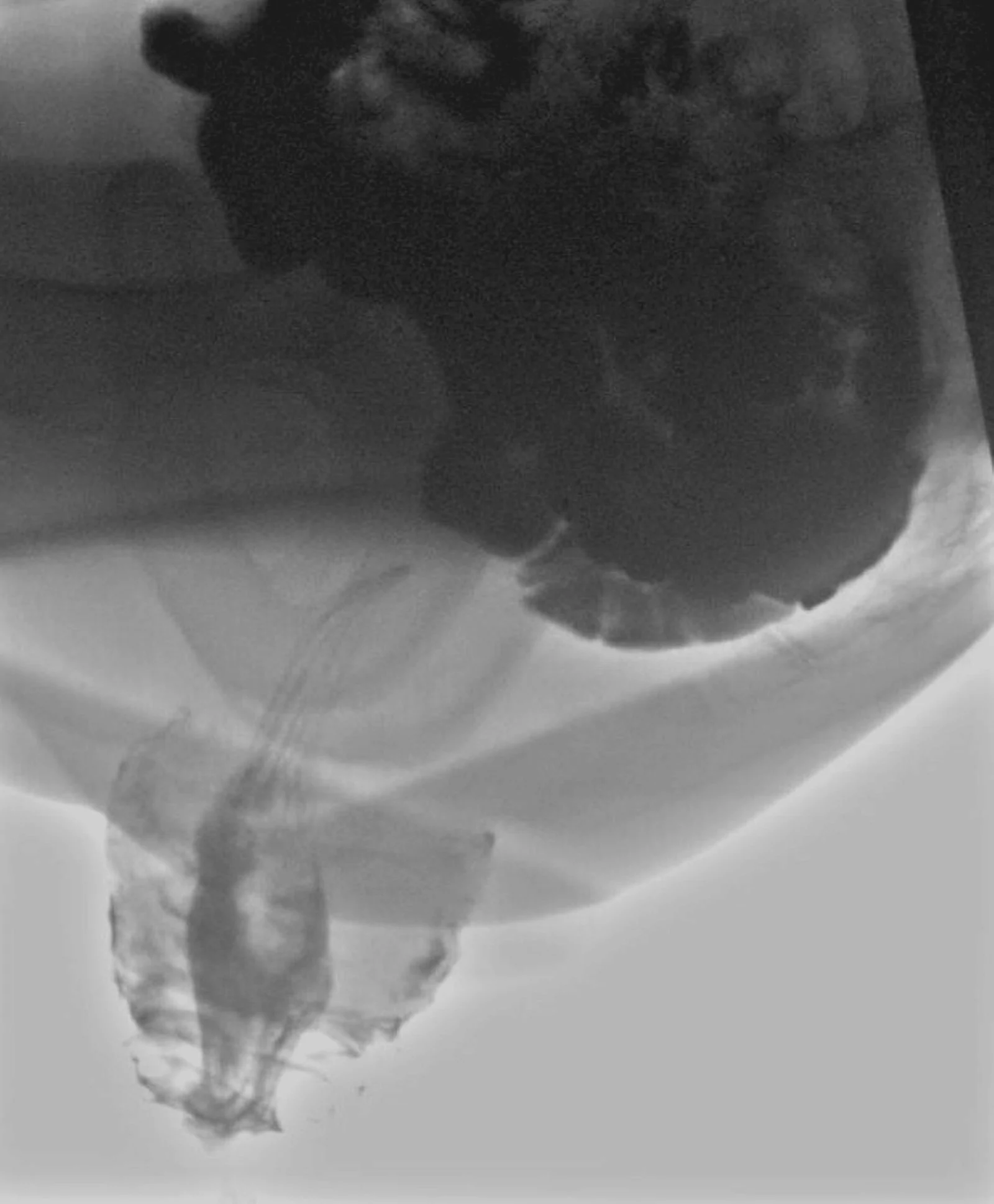
Defecating proctography demonstrating Grade IV RP. RP, rectal prolapse

Apart from a single episode of severe constipation two years previously, there was no documented history of chronic constipation or functional bowel disorder. At his colorectal review in May 2025, the patient described a full-thickness RP that had first developed following this isolated episode. This had subsequently been exacerbated by chronic straining to void in the context of ketamine-associated uropathy. He reported obstructed defecation symptoms, and physical examination confirmed a full-thickness prolapse. Defecating proctography was requested to further characterize this and demonstrated a Grade IV RP (Figure 2). In terms of definitive management, ventral mesh rectopexy was recommended, but with a caveat that if his straining behavior persisted postoperatively, recurrence was likely.

Despite input from the urology and pain teams, his suprapubic pain remained difficult to control. Intravesical local anesthetic instillation was attempted but not tolerated, and in September 2025, the patient relapsed for analgesic purposes. As straining precluded definitive surgical management of his prolapse, he was referred to a specialized pain service and listed for discussion at a regional multidisciplinary meeting for consideration of cystectomy. A pelvic MRI was performed in December 2025, revealing radiological features consistent with end-stage ketamine uropathy and associated proctitis.

Key clinical features and outcomes across both cases are summarized in Table 1.

**Table 1:** Summary of key clinical features and outcomes across both cases. MDT, multidisciplinary team; RP, rectal prolapse

Variable	Case 1	Case 2
Age	20	27
Sex	F	M
Ketamine use duration	Two years	Four years
Ketamine usage (amount)	Daily use (exact dose not documented)	1-2 g daily
Bladder capacity on cystoscopy	300 mL	100 mL
Upper urinary tract findings (imaging)	Normal renal tract ultrasound	Bilateral hydroureter and bilateral mild hydronephrosis on CT
Anorectal investigation(s)	Colonoscopy; anorectal manometry; defecating proctography	Defecating proctography
Anorectal pathology (Oxford classification)	Grade III (rectoanal intussusception) - confirmed on proctography	Grade IV (external prolapse) - confirmed on proctography
Initial management of RP	Conservative: laxatives, pelvic floor physiotherapy	Considered for ventral mesh rectopexy (surgery deferred)
Outcome/ongoing management	Poor compliance; discharged from pelvic floor service	Surgery deferred due to ongoing straining; MDT discussion for cystectomy; ongoing specialist pain and urology input

## Discussion

These case reports illustrate how two young adults with chronic ketamine misuse developed a spectrum of anorectal pathology, ranging from pelvic floor dyssynergia and rectoanal intussusception to established full-thickness RP. A consistent mechanistic pattern is observable across both cases. Severe ketamine-associated lower urinary tract dysfunction resulted in persistent suprapubic pain, reduced bladder capacity, and frequent straining to void. Importantly, neither patient had documented chronic constipation or preexisting functional bowel dysfunction preceding the onset of ketamine-associated uropathy. This repeated Valsalva-type effort across both cases represents a unifying behavioral and physiological insult to the pelvic floor, culminating in RP. Instead, persistent urinary straining represented the principal repetitive mechanical insult to the pelvic floor, with anorectal symptoms developing only after progression of pelvic floor pathology.

As summarized in Table 1, the severity of anorectal pathology appeared to parallel the degree of bladder dysfunction. Case 1, with comparatively less severe bladder dysfunction, developed functional pelvic floor dyssynergia and rectoanal intussusception without external prolapse. Case 2, however, had reduced functional bladder capacity and developed full-thickness prolapse. This gradation may support a dose-response hypothesis whereby prolonged and severe ketamine-associated bladder dysfunction amplifies the mechanical burden placed on the pelvic floor. It also illustrates how intussusception represents an intermediate stage in a continuum that may culminate in overt prolapse if the underlying precipitant persists.

While chronic straining to void provides a compelling unifying mechanism for the development of RP in the context of chronic ketamine misuse, other factors in these patients’ histories may have contributed to pelvic floor vulnerability.

The patient in the first case had documented psychiatric comorbidities: anxiety and depression. Psychiatric illness has previously been associated with RP in younger cohorts, potentially mediated by behavioral factors, altered pain perception, psychotropic medication effects, and/or coexisting functional bowel disorders [13,14]. Notably, inflammatory bowel disease in this patient was excluded based on endoscopic and histological findings. Solitary rectal ulcer syndrome was considered secondary to mechanical trauma from internal rectal intussusception.

In the second case, the prolapse initially followed an episode of severe constipation, indicating a preexisting pelvic floor insult. However, the subsequent exacerbation of prolapse in both cases correlated temporally with worsening ketamine-induced urinary dysfunction and straining behavior. This suggests that ketamine-associated uropathy may have acted as a propagating rather than initiating factor. As such, ketamine misuse in these cases should be viewed not necessarily as the sole etiological factor, but as a potent and potentially modifiable driver that amplified existing risk profiles for pelvic floor failure.

A particularly concerning observation across both cases was the development of a self-reinforcing cycle. Ketamine-induced bladder inflammation and fibrosis led to severe pain and reduced bladder capacity. Consequently, straining to void developed, thereby worsening pelvic floor integrity. Progressive prolapse then complicated surgical decision-making (Case 2), as ongoing straining precluded definitive RP repair. Additionally, repeated ketamine misuse by the patients for analgesic purposes compounded both urological and colorectal pathology. This highlights the difficulty of breaking the cycle once severe organ damage has occurred and underscores the importance of early recognition and sustained abstinence.

Nonetheless, the principal limitation of this study is its small sample size and observational design, which preclude establishing a definitive causal relationship between chronic ketamine misuse and the development of RP. Larger prospective studies incorporating standardized anorectal symptom assessment and longitudinal follow-up are required to validate this proposed mechanism.

## Conclusions

These cases suggest a plausible link between ketamine-induced urinary dysfunction, strained voiding patterns, and progressive pelvic floor failure in young adults. Although ketamine misuse may not represent the sole etiological factor, it appears to act as a potent and potentially modifiable driver that amplifies existing risk profiles for RP. Clinicians managing such patients with ketamine-associated uropathy should remain vigilant for emerging anorectal symptoms. Early intervention targeting straining behavior and sustained abstinence is critical to prevent irreversible pelvic floor prolapse. Equally, colorectal surgeons encountering RP in younger patients with concomitant lower urinary tract symptoms should inquire about recreational ketamine use at initial presentation.
